# Iron Deficiency in HbSC Disease Treated With Repetitive Phlebotomy Is Associated With Fewer Sickle Cell Disease‐Related Complications

**DOI:** 10.1002/ajh.70045

**Published:** 2025-09-04

**Authors:** Marissa J. M. Traets, Aida S. Kidane Gembremeskel, Jennifer Eijkelenboom‐Bos, Birgitte A. van Oirschot, Sigrid van der Veen, Henk Russcher, Wouter W. van Solinge, Mandy N. Lauw, Erfan Nur, Bart J. Biemond, Marjon H. Cnossen, Anita W. Rijneveld, Richard van Wijk, Eduard J. van Beers, Minke A. E. Rab

**Affiliations:** ^1^ Department of Central Diagnostic Laboratory—Research University Medical Center Utrecht, Utrecht University Utrecht the Netherlands; ^2^ Department of Pediatric Hematology and Oncology Erasmus MC Sophia Children's Hospital, University Medical Center Rotterdam Rotterdam the Netherlands; ^3^ Center for Benign Hematology, Thrombosis and Hemostasis—Van Creveldkliniek University Medical Center Utrecht Utrecht the Netherlands; ^4^ Department of Clinical Chemistry Erasmus MC, University Medical Center Rotterdam Rotterdam the Netherlands; ^5^ Department of Hematology Erasmus MC, University Medical Center Rotterdam Rotterdam the Netherlands; ^6^ Department of Hematology Amsterdam University Medical Center Amsterdam the Netherlands; ^7^ Department of Blood Cell Research Sanquin Research and Landsteiner Laboratory Amsterdam the Netherlands

**Keywords:** iron, sickle cell disease, phlebotomy

## Abstract

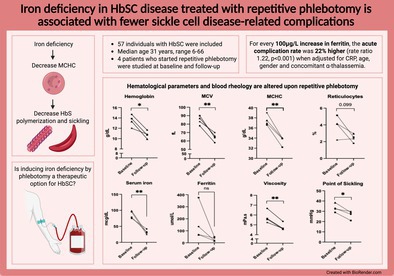

To the Editor,

1

Hemoglobin SC (HbSC) is the second most common form of sickle cell disease (SCD), resulting from compound heterozygosity for hemoglobin S (HbS) and hemoglobin C (HbC) [1]. Individuals with HbSC have higher hematocrit and blood viscosity levels compared to HbSS individuals due to a lower degree of hemolysis [2]. In addition, the presence of both HbS and HbC is associated with increased red blood cell (RBC) dehydration due to potassium and water efflux. This promotes RBC sickling by increasing HbS polymerization and decreasing RBC deformability, further contributing to increased blood viscosity [2]. Because the pathophysiology differs from that of HbSS, HbSC individuals require distinct therapeutic strategies. Aside from hydroxyurea, no other disease‐modifying therapies are available. Given the impact of the high blood viscosity and RBC dehydration, therapeutic interventions should focus on addressing these key pathophysiological features of HbSC disease. Iron is an essential element required to produce hemoglobin and RBCs. While iron deficiency is typically considered harmful and requires treatment in most cases, previous studies suggest it may have beneficial effects in SCD by lowering mean corpuscular hemoglobin concentration (MCHC), thus reducing the intracellular HbS concentration and delaying HbS polymerization [3, 4]. Retrospective studies have reported that repetitive phlebotomy induces iron deficiency and reduces blood viscosity, potentially decreasing the frequency of vaso‐occlusive episodes (VOE) in HbSC [3]. These findings suggest that inducing iron deficiency may be a promising therapeutic approach for individuals with HbSC [3, 4]. This study investigates the impact of iron deficiency on hematological characteristics, various functional properties of the RBC, and the history of SCD‐related complications in individuals with HbSC in a cross‐sectional cohort study and a longitudinal case series.

Adults and children (> 3 years of age) with HbSC at steady state, without recent blood transfusions (< 3 months), were eligible to participate. HbSC individuals who started repetitive phlebotomy were studied at baseline and follow‐up. Phlebotomy was performed every 2 weeks until hemoglobin levels reached 10.0 to 10.55 g/dL. Thereafter, the interval was prolonged depending on hemoglobin levels. Patient characteristics, as well as acute and chronic SCD‐related complications, were collected from medical charts. Whole blood viscosity was measured using a cone‐plate viscometer. Oxygen and osmotic gradient ektacytometry were performed to assess RBC deformability during varying oxygen or osmotic gradients. In vitro flow experiments assessed RBC adhesion to laminin using a microfluidic device. Statistical analysis was performed in R Studio and GraphPad Prism. Additional details are provided in the Supporting Information S1.

Fifty‐seven individuals with HbSC (median age 31 years, range 6–66) were included (Table S1). Both multivariable Poisson and logistic regression showed a significant correlation between ferritin levels and a history of acute complications (Table 1). For every 100 μg/L increase in ferritin, the acute complication rate was 22% higher (rate ratio: 1.22, *p* < 0.001) when adjusted for CRP, age, gender, and concomitant α‐thalassemia. When analyzing individual acute complications, a significant correlation was observed with VOEs in the multivariable Poisson regression model (rate ratio 1.16, *p* = 0.030) and complications that required acute exchange transfusion in the multivariable logistic regression model (OR 2.95, *p* = 0.038). No correlations between ferritin and chronic SCD‐related complications were observed. Similarly, no significant associations between transferrin saturation (TSAT) or serum iron and acute or chronic SCD‐related complications were found, although we observed a trend between TSAT and the occurrence of cerebral infarction (*p* = 0.080) and serum iron and ACS (*p* = 0.086; Tables S2 and S3). White blood cell and neutrophil count did not correlate with SCD‐related complications (data not shown). Next, we stratified individuals on low ferritin levels (≤ 20 μg/L, *n* = 7), normal ferritin levels (21–240 μg/L, *n* = 42), and high ferritin levels (> 240 μg/L, *n* = 8) to explore associations between ferritin and laboratory parameters (Figure S1). HbSC individuals with low ferritin levels were either men (*n* = 4) on repetitive phlebotomy or women (*n* = 3) in their reproductive period. Blood transfusions were administered to six HbSC individuals with normal ferritin levels, who received one or two erythrocyte transfusions in the 5 years before inclusion. In addition, two HbSC individuals with high ferritin levels underwent a single blood exchange transfusion and received two or four occasional transfusions in the 5 years before inclusion. HbSC individuals with low ferritin levels had significantly lower MCV, MCHC levels, reticulocyte count, and CRP levels. Serum iron and TSAT were decreased, consistent with low ferritin levels. Hemoglobin, hematocrit, bilirubin, LDH, and blood viscosity levels did not differ between HbSC individuals with low, normal, or high ferritin. Iron‐deficient HbSC individuals exhibited a pronounced leftward shift, indicating a reduced membrane surface area‐to‐volume ratio and increased dehydration (Figure S2A). Ferritin was not significantly associated with the sickling tendency (PoS) or RBC adhesion to laminin (Figures S2B and S3).

**TABLE 1:** Multivariable Poisson and logistic regression analysis of 100 μg/L increase in ferritin with SCD‐related complications in individuals with HbSC.

	100 μg/L increase in ferritin^a^					
	Univariable Poisson regression			Multivariable Poisson regression		
	RR	(95% CI)	*p*	RR	(95% CI)	*p*
Total complication rate	1.11	(1.01–1.21)	**0.020**	1.09	(1.00–1.17)	**0.043**
Acute complication rate	1.22	(1.09–1.35)	**< 0.001**	1.22	(1.09–1.36)	**< 0.001**
VOEs	1.15	(0.98–1.30)	**0.046**	1.16	(1.00–1.30)	**0.030**
Chronic complication rate	0.99	(0.81–1.15)	0.898	0.94	(0.76–1.11)	0.537

	Univariable logistic regression			Multivariable logistic regression		
	OR	(95% CI)	*p*	OR	(95% CI)	*p*
≥ 3 (Any) complications	1.79	(1.14–3.15)	**0.026**	2.09	(1.09–5.84)	0.086
≥ 2 Acute complications	2.15	(1.29–4.05)	**0.010**	3.90	(1.55–1.80)	**0.021**
VOE ≥ 2 < 24 months	1.33	(0.93–2.07)	0.136	1.58	(0.97–3.60)	0.176
Acute chest syndrome	2.35	(1.34–4.67)	**0.008**	2.19	(1.19–6.54)	0.072
Cerebral infarction	1.16	(0.69–1.72)	0.466	1.20	(0.67–1.89)	0.428
Acute exchange transfusion	2.05	(1.24–3.79)	**0.014**	2.95	(1.32–11.31)	**0.038**
≥ 2 Chronic complication	0.98	(0.55–1.44)	0.935	0.92	(0.50–1.37)	0.707
Retinopathy	0.91	(0.62–1.27)	0.584	0.81	(0.47–1.18)	0.357
Osteonecrosis	1.00	(0.63–1.42)	0.995	0.94	(0.58–1.37)	0.750
Chronic kidney disease	0.97	(0.55–1.40)	0.880	0.83	(0.39–1.32)	0.552
Cholelithiasis	1.23	(0.81–1.83)	0.269	1.13	(0.72–1.71)	0.533

*Note*: The average of three ferritin levels measured on separate dates during steady state was calculated and used for multivariable Poisson and logistic regression analysis. In the multivariable analysis, the variables were further adjusted for age, sex, and α‐thalassemia status. Acute complications include vaso‐occlusive episodes (VOEs), acute chest syndrome, cerebral infarction, or complications such as suspected bone marrow necrosis or fat embolic syndrome that required immediate exchange blood transfusion. Chronic complications included retinopathy, osteonecrosis, chronic kidney disease, and history of cholelithiasis. Bold values indicate statistical significance.

Abbreviations: OR, odds ratio; RR, rate ratio.

^a^ Ferritin values adjusted for CRP using the BRINDA inflammation adjustment method were used.

To explore how repetitive phlebotomy affects laboratory parameters and the occurrence of VOE, we followed four individuals (all male) who underwent repetitive phlebotomy because of high hemoglobin levels and SCD‐related complications, including VOEs, progressive retinopathy, or renal infarction (Table S4). Following repetitive phlebotomy, individuals experienced no or fewer VOEs over 11–188 months. Two individuals with progressive retinopathy showed no symptom progression after regular phlebotomy. Another individual with renal infarction showed improvement on computed tomography 1 year after starting repetitive phlebotomy. No treatment‐related adverse events, such as symptomatic anemia or hypotension, were reported after phlebotomy. Repetitive phlebotomy resulted in a median decrease in hemoglobin from 13.6 to 10.8 g/dL (*p* = 0.014) and hematocrit from 38.5% to 32.8% (*p* = 0.026, Figure 1). Furthermore, significant decreases in MCV and MCHC were observed. Iron deficiency occurred in all phlebotomized patients, with a median decrease in ferritin from 102 to 30 μg/L (*p* = 0.15), serum iron from 86.6 to 31.6 mcg/dL (*p* = 0.009), and TSAT from 24.5% to 6.5% (*p* = 0.011). In three patients, elevated percentage reticulocytes and LDH levels decreased following regular phlebotomy. Bilirubin levels decreased from 1.74 to 0.73 mg/dL; however, this finding was not statistically significant (*p* = 0.081). In addition, phlebotomy significantly reduced blood viscosity (from 5.6 to 4.6 mPa s, *p* = 0.007). The effect on the hematocrit‐to‐viscosity ratio was variable. Furthermore, phlebotomy increased the percentage of hypochromic RBCs and decreased the percentage of total dense RBCs, while the microcytic dense RBCs increased during phlebotomy (Figure S4). Regular phlebotomy resulted in a leftward shift of the curve in the hypo‐osmolar region with a significant decrease in O_min_, reflecting a reduced RBC membrane surface area‐to‐volume ratio compared to baseline. The maximum deformability (EI_max_) also decreased significantly while the O_max_ and O_hyper_ remained stable. Oxygen gradient ektacytometry parameters improved upon phlebotomy (Figure S5) with a significantly delayed onset of RBC sickling during deoxygenation (*p* = 0.030). Following phlebotomy, RBC adhesion to laminin decreased in three out of four individuals (Figure S5).

**FIGURE 1:**
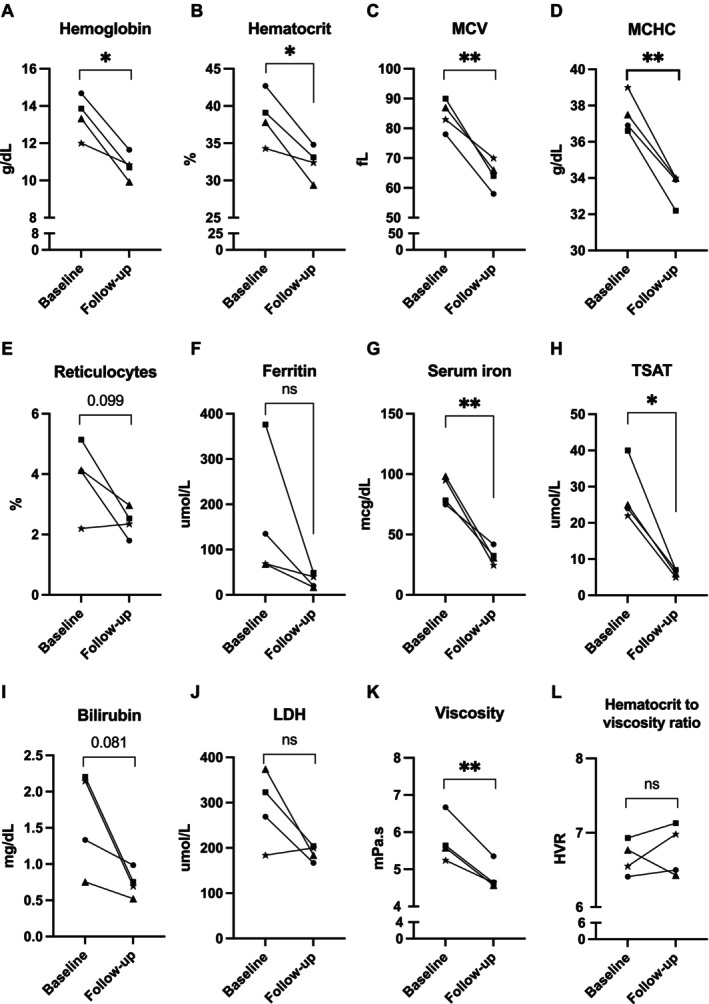
Hematological parameters and blood rheology are altered upon repetitive phlebotomy. LDH, lactate dehydrogenase; MCHC, mean corpuscular hemoglobin concentration; MCV, mean corpuscular volume; TSAT, transferrin saturation. *p < 0.05, **p < 0.01, ns = nonsignificant. *p* values showing a trend (*p* < 0.10) were reported as numerical values.

Our study indicates that low ferritin levels are associated with fewer acute SCD‐related complications in individuals with HbSC, a finding further supported by our data following repetitive phlebotomy. After adjusting for inflammation, as indicated by CRP levels, the association between ferritin and clinical outcomes in HbSC remained unchanged, indicating that ferritin as a marker of iron stores is associated with SCD‐related complications in HbSC. SCD patients who experience severe complications are often treated with transfusions. Patients on chronic transfusions were excluded to avoid confounding, as frequent transfusions can result in iron overload. Two HbSC individuals with high ferritin (329 and 807 μg/L) received occasional transfusions in the past 5 years. Their iron and TSAT levels (63.7/64.8 mcg/dL; 15%/19%) did not indicate iron overload. Moreover, exchange transfusion generally does not lead to iron overload [5]. Larger studies are needed to affirm the relationship between ferritin and acute SCD‐related complications in HbSC individuals.

Although the sample size to assess the effects of phlebotomy was small, our results suggest a potentially important role of phlebotomy in HbSC. Therapeutic phlebotomy has been practiced for decades, primarily in individuals with polycythemia and hereditary hemochromatosis. Recently, numerous studies have explored the application of phlebotomy in SCD [3, 6]. Phlebotomy may seem counterintuitive in anemic individuals, but the largest study on phlebotomy in SCD (64 patients) also demonstrated a reduction in acute event recurrence of 71% [6]. All studies investigating phlebotomy in SCD report on changes in hematological parameters and clinical complications. Our study is the first to also investigate various functional properties of the RBC reflecting sickling, RBC dehydration, and adhesion before and after phlebotomy. Most notably, phlebotomy led to a reduced tendency for sickling. Published studies on phlebotomy in SCD report a variation in targeted hemoglobin levels, phlebotomy intervals, and clinical endpoints, reflecting the absence of prospective randomized clinical trials and standardized guidelines. Due to this lack of standardization, phlebotomy practices are guided by expert opinion, leading to variability across healthcare centers. This highlights the need for randomized clinical studies to evaluate the role of phlebotomy in SCD. Aside from phlebotomy, a decrease in iron levels can also be achieved by pharmacological therapies, such as iron chelation or hepcidin administration. These practices are mainly applied to individuals with iron overload due to blood transfusions. Despite its potential benefits in SCD, iron deficiency is linked to impaired growth in children and cognitive impairment in both children and the elderly. Therefore, iron restriction or phlebotomy is not recommended for children or pregnant women. Nevertheless, phlebotomy remains a valuable option for inducing iron deficiency, particularly in HbSC disease, since it offers the added benefit of decreasing blood viscosity, a benefit not provided by pharmacological therapeutic approaches.

In conclusion, the data reported here suggest that a low serum ferritin is associated with fewer SCD‐related acute complications in individuals with HbSC. Patient‐tailored treatment strategies need to take into account the unique rheology of HbSC disease. Phlebotomy may be a particularly effective therapy in HbSC, as it can improve blood rheology by reducing blood viscosity and iron levels. These promising results warrant further prospective studies on phlebotomy as a low‐risk and low‐cost therapy to reduce the disease burden for HbSC.

## Author Contributions

M.J.M.T., R.v.W., and M.A.E.R. designed the study. M.J.M.T., J.E.‐B., and B.A.v.O. developed laboratory methodology and performed laboratory experiments. M.J.M.T., A.S.K.G., S.v.d.V., M.N.L., E.N., B.J.B., M.H.C., A.W.R., E.J.v.B., and M.A.E.R. recruited and included patients. H.R. supported the collection of laboratory parameters. M.J.M.T. collected clinical data, performed the analysis, and wrote the manuscript. R.v.W. and M.A.E.R. supervised the research. All authors revised the manuscript and approved the final version.

## Conflicts of Interest

M.J.M.T. received research funding from Agios Pharmaceuticals Inc. A.S.K.G. is supported by a grant from “Het Sikkelcelfonds.” M.N.L. received research funding from ZonMW, LeoPharma, and INVENT‐VTE; reports consultancy fees (all to institute) from BMS/Pfizer, Amgen, Inari, Viatris, Servier, and AbbVie. E.N. received research funding from Novartis; reports a consulting role with Novartis and Vertex. B.J.B. received research funding from Sanquin, BMS, Pfizer, and Novartis; received honoraria from Sanofi; reports advisory board participation with Celgene, CSL Behring, Pfizer, and Novo Nordisk. A.W.R. received honoraria from Pfizer, Vertex Pharmaceuticals, BMS, and Servier. R.v.W. received research funding from Agios Pharmaceuticals Inc. and Pfizer; reports a consulting role with Agios Pharmaceuticals Inc., Pfizer, and RR Mechatronics. E.J.v.B. received research funding from Agios Pharmaceuticals Inc.; reports a consulting role with Agios Pharmaceuticals Inc. and Pfizer. M.A.E.R. received research funding from Agios Pharmaceuticals Inc. and Pfizer; reports a consulting role with Agios Pharmaceuticals Inc., Vertex Pharmaceuticals, and RR Mechatronics. The other authors declare no conflicts of interest.

## Supporting information


**Data S1:** Supporting Information.

**Data S2:** Supporting Information.

## Data Availability

Data and protocols are available upon request (email m.a.e.rab@umcutrecht.nl). Data will be shared as is compliant with the General Data Protection Regulation and European Union privacy laws.
