# Supplementary material for: Iron Deficiency in HbSC Disease Treated With Repetitive Phlebotomy Is Associated With Fewer Sickle Cell Disease‐Related Complications
Source: Am J Hematol. 2025 Sep 4;100(11):2123–7. doi: 10.1002/ajh.70045 (PMC12516645; doi:10.1002/ajh.70045)
Supplement: Supplementary file 1 — Data S1: Supporting Information. [file AJH-100-2123-s002.pdf]

Supplemental Methods

Blood collection

Adults and children (>3 years of age) with HbSC from Dutch outpatient clinics in the Sickle Cell Outcome Research (SCORE) consortium in The Netherlands (Erasmus University Medical Center [UMC], Amsterdam UMC and Utrecht UMC) at steady state were eligible to participate. EDTA blood was collected of individuals in a steady state condition (i.e. not during a hospital admission and/or VOE) after written informed consent was obtained. Study procedures were approved by the local medical ethical committees (protocol number: 17/392) in accordance with the Declaration of Helsinki.

SCD-related complications

Patient characteristics and SCD-related complications were collected by reviewing the medical charts. VOEs were defined as acute episodes of pain, with no other medical underlying cause that resulted in a visit to a medical facility (e.g. hospital admission, emergency department or outpatient clinic visit). Acute complications included VOEs, ACS, cerebral infarction (ischaemic stroke or silent cerebral infarct) or complications, such as suspected bone marrow necrosis or fat embolic syndrome, that required immediate exchange blood transfusion. Episodes of priapism were excluded. Chronic complications included retinopathy (proliferative and non-proliferative), osteonecrosis, chronic kidney disease (CKD), leg ulcers and history of cholelithiasis. CKD was defined by the urine albumin to creatinine ratio (ACR, >3.4 mg/mmol) combined with the estimated glomerular filtration rate (eGFR) using the CKD-EPI formula.[1] Only VOEs that occurred within two years prior to blood collection were included. All other complications over the patient's lifetime were collected. A composite score for acute, chronic and total complications was created by summing the various types of SCD-related complications experienced throughout the patient's life, with a maximum of one point per complication type (e.g. two VOEs in the past 24 months would equal one point, two ACS throughout the patient's life would also equal one point).

Laboratory parameters

Routine hematological laboratory parameters were measured using the Cell-Dyn Sapphire hematology analyzer (Abbott Diagnostics). The percentage of total hypochromic RBCs (MCHC <28 g/dL and mean corpuscular volume [MCV] <120 fL), as well as total dense RBCs (MCHC >41 g/dL and MCV <120 fL) were obtained using the Advia 120/2120 hematology analyzer (Siemens Healthcare diagnostics). Additionally, microcytic dense RBCs (MCHC >41 g/dL and MCV <60 fL) were also measured by the Advia. High-performance liquid chromatography was performed to analyze the levels of normal hemoglobin (HbA), fetal hemoglobin (HbF), HbS and HbC (Tosoh G8). Ferritin, serum iron, transferrin saturation (TSAT), bilirubin, lactate dehydrogenase (LDH), and C-reactive protein (CRP) levels were measured on routine chemistry analyzers.

Blood viscosity

Whole blood viscosity was measured at 37°C at a shear rate of 150 s^{-1} using a cone-plate viscometer (Brookfield DVNextLV, spindle CPA-40Z). Hematocrit to viscosity ratio (HVR), representing oxygen delivery efficiency, was calculated by dividing hematocrit by viscosity.

Oxygen and osmotic gradient ektacytometry

Oxygen and osmotic gradient ektacytometry (oxygenscan and osmoscan, respectively) were performed to measure RBC deformability, expressed as the elongation index (EI), by the Laser-Optical Rotational Red Cell Analyzer (Lorrca, RR Mechatronics) as previously described.[2–4] Oxygen gradient ektacytometry measures RBC deformability upon deoxygenation and reflects sickling behavior of RBCs by means of point of sickling (PoS).[2] The PoS is the partial pressure of oxygen (pO_2) at which the maximum EI (EI_{max}) decreases by 5% in response to deoxygenation. The minimum EI (EI_{min}) represents the minimal RBC deformability upon deoxygenation. Osmotic gradient ektacytometry measures RBC deformability under an osmotic gradient.[3,4] Parameters generated by osmotic gradient ektacytometry include the osmolality at minimum EI in the hypo-osmolar region (O_{min}), osmolality at maximum EI (O_{max}), maximum deformability (EI_{max}), and the osmolality at 50% of the EI_{max} in the hyper-osmolar region (O_{hyper}). The O_{min} represents the membrane surface area-to-volume ratio of RBCs, while the O_{hyper} represents the RBC hydration status.

RBC adhesion to laminin assay

In vitro flow experiments were performed to assess RBC adhesion to laminin using a microfluidic device (IBIDI µ-Slide I 0.4 uncoated). IBIDI slides were coated with 6.7 µg/mL laminin (BioLamina) for 2 hours at 37°C. Whole blood with a standardized concentration of $64x10^6$ RBCs per mL was diluted in HEPES buffer (132mM NaCl, 20 mM HEPES, 6 mM KCl, 1.2 mM K_2HPO_4, 1 mM $MgSO_4$, 1 mM $CaCl_2$, 5.55 mM glucose and 0.5% BSA, final pH 7.40 at room temperature). Diluted RBCs were perfused at room temperature at a shear stress of 0.5 dyn/cm^2 using a syringe pump (Harvard Apparatus PHD Ultra). After a continuous flow for 10 minutes, non-adherent RBCs were washed away by perfusing HEPES buffer for two minutes. Images were taken at nine different places using the Zeiss Observer Z1 microscope (20x objective). The number of adherent RBCs was quantified using ImageJ (Fiji).

Statistical analysis

Statistical analysis was performed in R Studio (v4.4.0) and GraphPad Prism (v10.4.1). Continuous laboratory parameters were presented as the median with interquartile range (IQR). Multivariable Poisson regression analysis was performed to calculate the rate ratio of ferritin in relation to the complication rate. A Quasi-Poisson analysis was performed when overdispersion was detected (deviance ratio >1.0). Logistic regression analysis was performed to explore associations of ferritin with SCD-related complications. The average of three ferritin levels measured on separate dates during steady state, was calculated and used for multivariable Poisson and logistic regression analysis. Ferritin levels were adjusted for CRP using the BRINDA (Biomarkes Reflecting Inflammation and Nutritional Determinants of Anemia) inflammation adjustment method[5], and the adjusted values were incorporated into the Poisson and logistic regression analyses. Both multivariable Poisson and logistic regression analyses were further adjusted for age, sex and α-thalassemia status. A Spearman rank correlation coefficient was used for the correlation between ferritin and laboratory parameters. To further explore the role of ferritin on RBC characteristics, the HbSC individuals were stratified into three groups with low (≤20 µg/L), normal (21-240 µg/L) and high ferritin (>240 µg/L) levels. For this analysis, the follow-up measurement of individuals on repetitive phlebotomy was used. Kruskal-Wallis test with Dunn's multiple comparisons test (continuous variables) was performed to compare the three groups. A p-value ≤0.05 was considered statistically significant.

References

1. Vaidya SR, Aeddula NR. Chronic Kidney Disease. *The Scientific Basis of Urology, Second Edition*. Published online July 31, 2024:257-264. doi:10.51249/hs.v4i01.1865
2. Rab MAE, van Oirschot BA, Bos J, et al. Rapid and reproducible characterization of sickling during automated deoxygenation in sickle cell disease patients. *Am J Hematol*. 2019;94(5):575-584. doi:10.1002/AJH.25443
3. Da Costa L, Suner L, Galimand J, et al. Diagnostic tool for red blood cell membrane disorders: Assessment of a new generation ektacytometer. *Blood Cells Mol Dis*. 2016;56(1):9-22. doi:10.1016/J.BCMD.2015.09.001
4. Lazarova E, Gulbis B, Oirschot B Van, Van Wijk R. Next-generation osmotic gradient ektacytometry for the diagnosis of hereditary spherocytosis: interlaboratory method validation and experience. *Clin Chem Lab Med*. 2017;55(3):394-402. doi:10.1515/CCLM-2016-0290
5. Namaste SM, Aaron GJ, Varadhan R, Peerson JM, Suchdev PS. Methodologic approach for the Biomarkers Reflecting Inflammation and Nutritional Determinants of Anemia (BRINDA) project. *Am J Clin Nutr*. 2017;106(Suppl 1):333S. doi:10.3945/AJCN.116.142273
