# Supplementary material for: Iron Deficiency in HbSC Disease Treated With Repetitive Phlebotomy Is Associated With Fewer Sickle Cell Disease‐Related Complications
Source: Am J Hematol. 2025 Sep 4;100(11):2123–7. doi: 10.1002/ajh.70045 (PMC12516645; doi:10.1002/ajh.70045)
Supplement: Supplementary file 2 — Data S2: Supporting Information. [file AJH-100-2123-s001.pdf]

Supplemental Results

Overview

Supplemental Table 1. HbSC patient characteristics

Supplemental Table 2. Multivariable Poisson and logistic regression analysis of 10-unit (%) increase in transferrin saturation was not correlated with SCD-related complications in individuals with HbSC

Supplemental Table 3. Multivariable Poisson and logistic regression analysis of 10-unit (mcg/dL) increase in serum iron was not correlated with SCD-related complications in individuals with HbSC

Supplemental Table 4. Patient characteristics of the four individuals on repetitive phlebotomy

Supplemental Figure 1. Low ferritin levels are associated with hematological and blood rheology parameters

Supplemental Figure 2. HbSC individuals with low ferritin levels have a decreased O_{min}, O_{max} and O_{hyper} in osmotic gradient ektacytometry, and decreased EI_{max} in oxygen gradient ektacytometry

Supplemental Figure 3. RBC adhesion to laminin is not affected by ferritin levels or repetitive phlebotomy

Supplemental Figure 4. Phlebotomy increased % hypochromic and decreased dense RBCs, except for microcytair dense RBCs

Supplemental Figure 5. Osmotic and oxygen gradient ektacytometry after repetitive phlebotomy

Supplemental Table 1. HbSC patient characteristics

	HbSC cohort (n=57)
**Patient characteristics**	
Age, years	31 (6-66)
Adults (≥ 18 years), n (%)	45 (78.9)
Sex, female, n (%)	25 (43.9)
Hydroxyurea, n (%)	10 (17.5)
α-thalassemia, n (%)	
αα/α-	19 (33.3)
αα/αα	37 (64.9)
Unknown	1 (1.8)
**Laboratory characteristics**	
Hemoglobin (g/dL)*	12.0 (8.8-15.0)
Hemoglobin F (%)	1.6 (0.0-14.2)
Hemoglobin S (%)	44.1 (37.5-45.9)
Hemoglobin C (%)	44.8 (36.2-46.8)
Ferritin (µg/L)*	108 (8-807)
TSAT (%)*	21 (6-49)
**SCD-related complications**	
No complications, n (%)	8 (14.0)
Total complication rate	2 (0-7)
≥2 acute complications, n (%)	10 (17.5)
≥2 VOE <24m, n (%)	12 (21.1)
Acute chest syndrome, n (%)	8 (14.0)
Cerebral infarction, n (%)	6 (10.5)
Complication that required acute exchange transfusion, n (%)	10 (17.5)
≥2 chronic complications, n (%)	10 (17.5)
Retinopathy, n (%)	29 (50.9)
Osteonecrosis, n (%)	14 (24.6)
Chronic kidney disease, n (%)	11 (19.3)
Leg ulcer, n (%)	0 (0.0)
Cholelithiasis, n (%)	8 (14.0)

*Reference interval hemoglobin 11.9-17.2 g/dL, ferritin 20-240 µg/L, and transferrin saturation 20-45%
Numbers represent median (range), unless stated otherwise. Abbreviations: TSAT, transferrin saturation; VOE, vaso-occlusive episode;

Supplemental Table 2. Multivariable Poisson and logistic regression analysis of 10-unit (%) increase in transferrin saturation was not correlated with SCD-related complications in individuals with HbSC

	10-unit (%) increase in TSAT					
	Univariable Poisson regression			Multivariable Poisson regression		
	RR	(95% CI)	P-value	RR	(95% CI)	P-value
**Total complication rate**	1.01	(0.80-1.27)	0.925	1.03	(0.83-1.27)	0.790
**Acute complication rate**	1.09	(0.73-1.56)	0.666	1.23	(0.82-1.80)	0.315
VOEs	1.11	(0.75-1.59)	0.576	1.14	(0.77-1.67)	0.496
**Chronic complication rate**	0.86	(0.61-1.17)	0.348	0.89	(0.61-1.26)	0.514
	**Univariable logistic regression**			**Multivariable logistic regression**		
	**OR**	**(95% CI)**	**P-value**	**OR**	**(95% CI)**	**P-value**
**≥3 (any) complications**	0.89	(0.45-1.68)	0.727	0.85	(0.37-1.84)	0.693
**≥2 acute complications**	1.24	(0.58-2.57)	0.556	2.11	(0.75-6.55)	0.161
VOE ≥2 <24 months	1.05	(0.50-2.08)	0.899	1.03	(0.38-2.70)	0.956
Acute chest syndrome	1.00	(0.89-1.09)	0.932	3.63	(0.73-2.97)	0.141
Cerebral infarction	2.17	(0.91-5.40)	0.078	4.48	(1.01-3.41)	0.080
Acute exchange transfusion	1.03	(0.46-2.15)	0.930	1.15	(0.38-3.37)	0.791
**≥2 chronic complication**	0.74	(0.29-1.60)	0.412	0.85	(0.29-2.23)	0.746
Retinopathy	0.90	(0.49-1.63)	0.733	0.91	(0.46-1.77)	0.776
Osteonecrosis	0.82	(0.38-1.61)	0.580	0.84	(0.34-1.89)	0.675
Chronic kidney disease	0.96	(0.43-1.97)	0.923	0.74	(0.24-2.00)	0.566
Cholelithiasis	1.44	(0.64-3.15)	0.357	2.09	(0.62-9.43)	0.260

The variables were adjusted for age, sex, α-thalassemia status and C-reactive protein in the multivariable analysis. Acute complications include vaso-occlusive episodes (VOEs), acute chest syndrome, cerebral infarction or complications, such as suspected bone marrow necrosis or fat embolic syndrome, that required immediate exchange blood transfusion. Chronic complications included retinopathy, osteonecrosis, chronic kidney disease and history of cholelithiasis. Abbreviations: RR, rate ratio; OR, odds ratio.

Supplemental Table 3. Multivariable Poisson and logistic regression analysis of 10-unit (mcg/dL) increase in serum iron was not correlated with SCD-related complications in individuals with HbSC

	10-unit (mcg/dL) increase in serum iron					
	Univariable Poisson regression			Multivariable Poisson regression		
	RR	(95% CI)	P-value	RR	(95% CI)	P-value
**Total complication rate**	1.04	(0.97-1.11)	0.268	1.03	(0.97-1.09)	0.346
**Acute complication rate**	1.04	(0.93-1.16)	0.440	1.05	(0.94-1.17)	0.369
VOEs	1.05	(0.94-1.16)	0.345	1.03	(0.91-1.14)	0.652
**Chronic complication rate**	1.01	(0.92-1.10)	0.759	1.01	(0.92-1.11)	0.778
	**Univariable logistic regression**			**Multivariable logistic regression**		
	**OR**	**(95% CI)**	**P-value**	**OR**	**(95% CI)**	**P-value**
**≥3 (any) complications**	1.00	(0.81-1.20)	0.967	0.96	(0.75-1.19)	0.691
**≥2 acute complications**	1.09	(0.87-1.36)	0.405	1.18	(0.88-1.59)	0.247
VOE ≥2 <24 months	1.06	(0.86-1.30)	0.556	0.97	(0.73-1.29)	0.853
Acute chest syndrome	1.07	(0.83-1.35)	0.556	1.72	(0.99-3.60)	0.086
Cerebral infarction	1.29	(1.01-1.70)	**0.046**	1.47	(0.94-2.68)	0.133
Acute exchange transfusion	1.05	(0.83-1.30)	0.653	1.03	(0.74-1.38)	0.864
**≥2 chronic complication**	1.02	(0.79-1.26)	0.884	1.05	(0.80-1.37)	0.694
Retinopathy	1.06	(0.89-1.28)	0.516	1.10	(0.90-1.37)	0.350
Osteonecrosis	1.00	(0.81-1.22)	0.988	0.97	(0.75-1.21)	0.779
Chronic kidney disease	1.08	(0.86-1.3)	0.490	0.99	(0.72-1.29)	0.928
Cholelithiasis	1.20	(0.95-1.52)	0.117	1.29	(0.93-2.10)	0.191

The variables were adjusted for age, sex, α-thalassemia status and C-reactive protein in the multivariable analysis. Acute complications include vaso-occlusive episodes (VOEs), acute chest syndrome, cerebral infarction or complications, such as suspected bone marrow necrosis or fat embolic syndrome, that required immediate exchange blood transfusion. Chronic complications included retinopathy, osteonecrosis, chronic kidney disease and history of cholelithiasis. Abbreviations: RR, rate ratio; OR, odds ratio.

Supplemental Table 4. Patient characteristics of the four individuals on repetitive phlebotomy

	Patient 1	Patient 2	Patient 3	Patient 4
**Patient characteristics**	Male, 19y	Male, 26y	Male, 29y	Male, 48y
**Indication repetitive phlebotomy**	Progressive retinopathy grade 3	3 VOEs in past 9 months, osteonecrosis	Renal infarction (on CT) with albuminuria and macroscopic hematuria	Retinopathy, and sudden loss of vision due to PION
**Follow-up measurements**	8 months (before 8th phlebotomy)	5 months (before 6th phlebotomy)	7 months (before 7th phlebotomy)	8 months (before 10th phlebotomy)
**Duration of clinical follow-up**	18 months	12 months	12 months	11 months
**Clinical symptoms after repetitive phlebotomy**	Retinopathy stable, no VOEs	1 VOE	Improvement renal parenchym (on CT), no VOEs	Still loss of vision, no VOEs

Abbreviations: VOE, vaso-occlusive episode; CT, computed tomography; PION, posterior ischemic optic neuropathy.

Supplemental Figure 1. Low ferritin levels are associated with hematological and blood rheology parameters

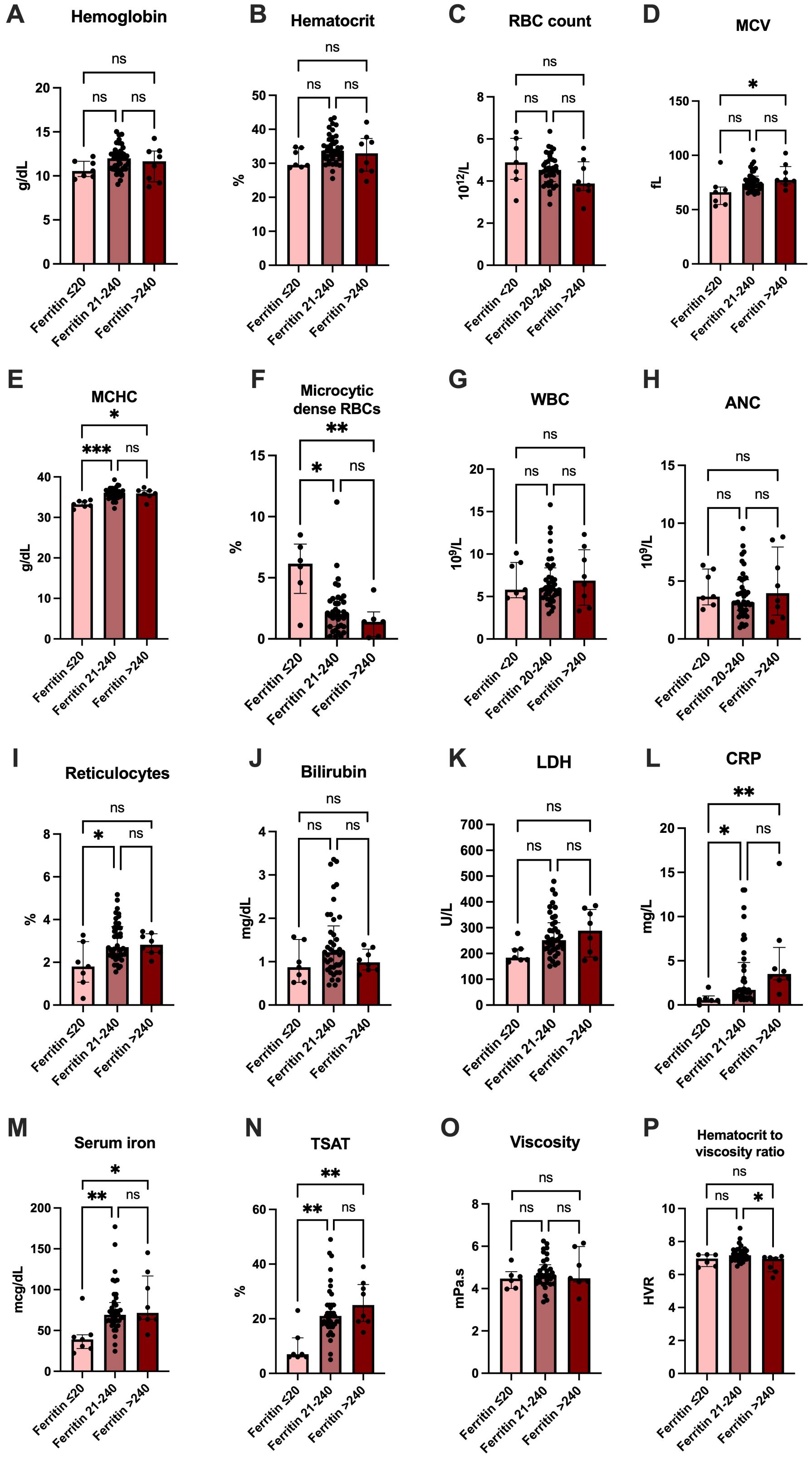

* p<0.05, **p<0.01, ***p<0.001, ns = non-significant. Kruskal-Wallis test with Dunn's multiple comparisons test was performed. Abbreviations: ANC, absolute neutrophil count; CRP, C-reactive protein; LDH, lactate dehydrogenase; MCV, mean corpuscular volume; MCH, mean corpuscular hemoglobin; RBCs, red blood cells; TSAT, transferrin saturation; WBC, white blood cell count.

Supplemental Figure 2. HbSC individuals with low ferritin levels have a decreased O_{min}, O_{max} and O_{hyper} in osmotic gradient ektacytometry, and decreased EI_{max} in oxygen gradient ektacytometry

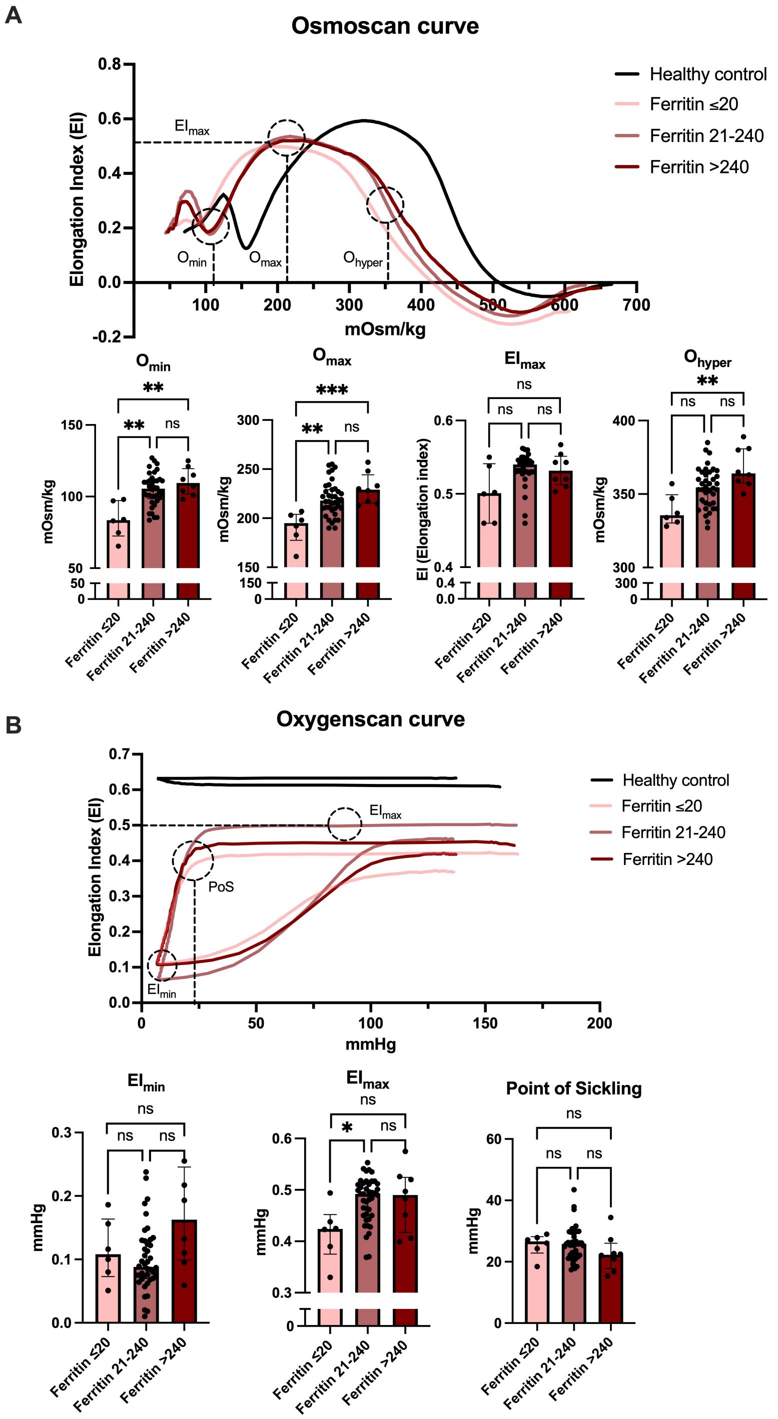

* $p<0.05$, ** $p<0.01$, *** $p<0.001$, ns = non-significant. Kruskal-Wallis test with Dunn's multiple comparisons test was performed. Representative osmoscan curves of a individuals with low, normal and high ferritin levels. (A) Individuals with ferritin ≤20 µg/L have a left-shifted osmoscan curve with a significant decrease in O_{min}, O_{max} and O_{hyper} indicating a reduced membrane surface area-to-volume ratio and more RBC dehydration. The maximum deformability (EI_{max}) was not significantly altered in individuals with low or high ferritin levels in the osmoscan. (B) Oxygen gradient ektacytometry showed a significant lower EI_{max} in individuals with low ferritin levels. EI_{min} and sickling tendency (reflected by the Point of Sickling) was not different between groups.

Supplemental Figure 3. RBC adhesion to laminin is not affected by ferritin levels or repetitive phlebotomy

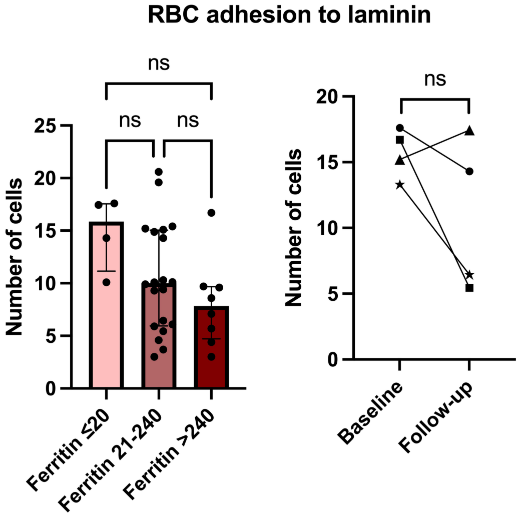

Abbreviations: RBC, red blood cell; ns, non-significant.

Supplemental Figure 4. Phlebotomy increased % hypochromic and decreased dense RBCs, except for microcytair dense RBCs

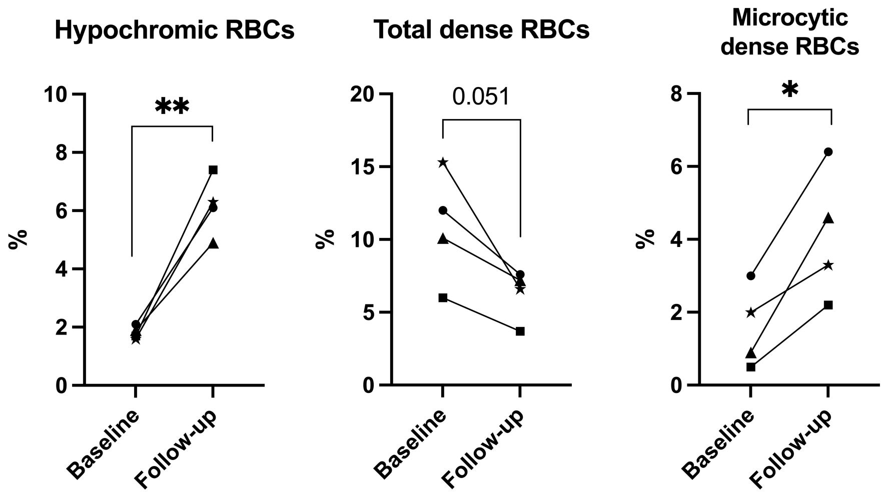

* p<0.05, **p<0.01. P-values showing a trend (p<0.10) were reported as numerical values.
Abbreviation: RBCs, red blood cells.

Supplemental Figure 5. Osmotic and oxygen gradient ektacytometry after repetitive phlebotomy

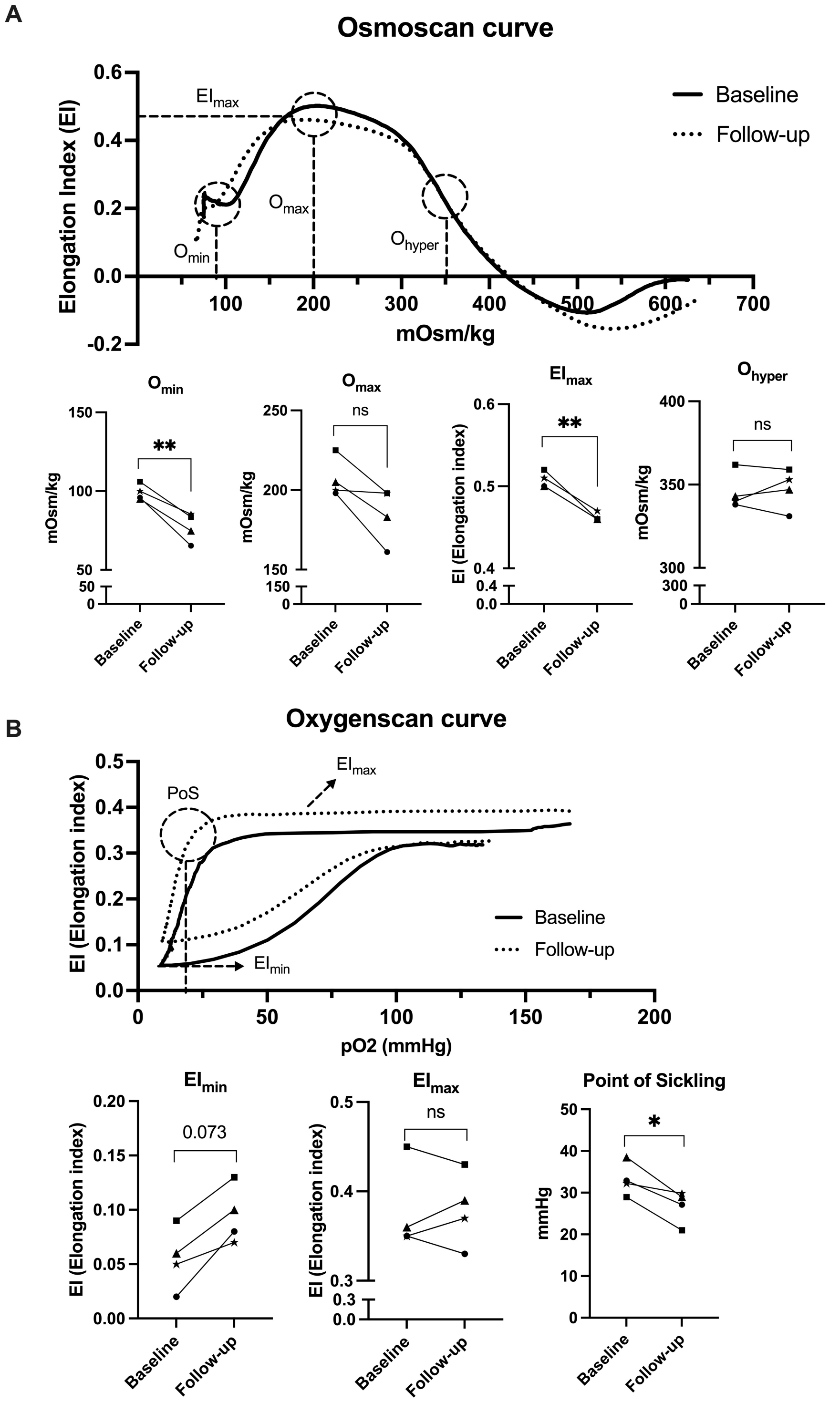

* p<0.05, **p<0.01, ns = non-significant. P-values showing a trend (p<0.10) were reported as numerical values.

Upon phlebotomy the osmoscan shifted to the left with a decrease in O_{min} (reflecting the membrane surface area-to-volume ratio), O_{max} (osmolality at EI_{max}) and EI_{max} (maximum deformability). The O_{hyper}, reflecting RBC hydration, was not affected. The oxygenscan showed a decrease in the EI_{min} (non-significant) and point of sickling (significant), indicating improved sickling tendency. Abbreviations: EI, elongation index.
